# Tissue losses and metabolic adaptations both contribute to the reduction in resting metabolic rate following weight loss

**DOI:** 10.1038/s41366-022-01090-7

**Published:** 2022-02-18

**Authors:** Alexandra Martin, Darius Fox, Chaise A. Murphy, Hande Hofmann, Karsten Koehler

**Affiliations:** 1 Department of Sport and Health Sciences, Technical University Munich, Munich, Germany; 2 Department of Nutrition and Health Sciences, University of Nebraska-Lincoln, Lincoln, NE USA

**Keywords:** Homeostasis, Fat metabolism

## Abstract

**Objective:**

To characterize the contributions of the loss of energy-expending tissues and metabolic adaptations to the reduction in resting metabolic rate (RMR) following weight loss.

**Methods:**

A secondary analysis was conducted on data from the *Comprehensive Assessment of Long-term Effects of Reducing Intake of Energy* study. Changes in RMR, body composition, and metabolic hormones were examined over 12 months of calorie restriction in 109 individuals. The contribution of tissue losses to the decline in RMR was determined by weighing changes in the size of energy-expending tissues and organs (skeletal muscle, adipose tissue, bone, brain, inner organs, residual mass) assessed by dual-energy X-ray absorptiometry with their tissue-specific metabolic rates. Metabolic adaptations were quantified as the remaining reduction in RMR.

**Results:**

RMR was reduced by 101 ± 12 kcal/d as participants lost 7.3 ± 0.2 kg (both *p* < 0.001). On average, 60% of the total reduction in RMR were explained by energy-expending tissues losses, while 40% were attributed to metabolic adaptations. The loss of skeletal muscle mass (1.0 ± 0.7 kg) was not significantly related to RMR changes (*r* = 0.14, *p* = 0.16), whereas adipose tissue losses (7.2 ± 3.0 kg) were positively associated with the reduction in RMR (*r* = 0.42, *p* < 0.001) and metabolic adaptations (*r* = 0.31, *p* < 0.001). Metabolic adaptations were correlated with declines in leptin (*r* = 0.27, *p* < 0.01), triiodothyronine (*r* = 0.19, *p* < 0.05), and insulin (*r* = 0.25, *p* < 0.05).

**Conclusions:**

During weight loss, tissue loss and metabolic adaptations both contribute to the reduction in RMR, albeit variably. Contrary to popularly belief, it is not skeletal muscle, but rather adipose tissue losses that seem to drive RMR reductions following weight loss. Future research should target personalized strategies addressing the predominant cause of RMR reduction for weight maintenance.

## Introduction

Worldwide obesity has tripled in the last decades, with more than 1.9 billion and 650 million adults considered overweight and obese, respectively [1]. With even modest weight reductions eliciting health improvements [2, 3], weight loss via the induction of a negative energy balance is encouraged for obesity treatment. Calorie restriction is the most common method for weight loss [4], and while initially efficacious, prolonged calorie restriction results in attenuated weight loss [5]. This weight loss attenuation occurs because of reductions in total daily energy expenditure (TDEE) that oppose the initial energy deficit [6]. These reductions in TDEE result in a return to energy balance at a lower level, which increases the likelihood of an energy surplus once weight loss efforts have stopped and predisposes individuals to future weight regain [7].

Although reductions secondary to weight loss have been reported for most components of TDEE [8], reductions in resting metabolic rate (RMR) have manifested most consistently [9, 10]. RMR is defined as the energy expended at rest for physiological functionality and comprises ~60–70% of TDEE in the normal population [11, 12], representing the largest contributor to TDEE. Thus, its preservation during weight loss has been targeted as a potential strategy to prevent the compensatory reductions in TDEE and subsequent weight regain [13].

It has been traditionally assumed that RMR preservation is enhanced when fat-free mass (FFM) is maintained during weight loss as FFM is considered the primary determinant of RMR [14]. Further, FFM losses can account for up to 50% of total weight loss [15] and thus may at least partially explain the variability observed in RMR following weight loss. However, FFM is a heterogeneous tissue [16], and the extent of the RMR reduction due to FFM loss is largely driven by the size and metabolic activity of the specific tissues that are lost. The brain and other vital organs consume more energy than resting skeletal muscle and bone when expressed relative to their size [16], yet FFM losses during weight loss are typically limited to skeletal muscle while the vital organs are preserved [17]. Thus, failure to account for the specific organ composition of FFM loss may result in misestimating RMR reductions due to tissue losses. Further, the contribution of reductions in other tissues outside of FFM secondary to weight loss should be accounted for as well [18]. Although fat mass, or more specifically adipose tissue, is considered to be relatively inert when compared to other tissues and organs [16], it is typically lost in much greater quantities [15] and may still meaningfully contribute to RMR reductions.

Yet, even when the contributions of organs and tissues are accounted for, RMR continues to decline beyond what would be expected based on the loss of energy-expending tissues. Müller et al. reported that only about one-third of the RMR reduction following 3 weeks of calorie-restricted weight loss was accounted for by metabolically active tissue, leaving two-thirds of RMR changes unexplained [19]. This unexplained portion is understood as a reduction in the metabolic activity of the existing remaining tissues [20], as evident by the close relationship between adaptive reductions in RMR and changes in key hormones involved in energy sensing and metabolism, such as leptin and thyroid hormones [20–23]. These metabolic adaptations represent the second important contributor to the reduction in RMR following weight loss and have been observed in prospective studies involving calorie restriction [8, 19] as well as in cross-sectional observations in populations with prolonged exposures to chronic energy deficiency [22, 24].

While both of these distinct phenomena—the loss of energy-expending tissues and the reduction in the metabolic activity of the remaining tissues—contribute to RMR reduction, it is unclear whether each contributor occurs independently or whether the magnitude of different tissue losses impacts the extent of RMR reductions and metabolic adaptations. The purpose of the present analysis was to quantify the unique contribution of these two components to RMR reduction during prolonged weight loss in healthy normal weight and overweight individuals and their relationship with each other. To address this objective, we retrospectively analyzed data from the Comprehensive Assessment of Long-term Effects of Reducing Intake of Energy (CALERIE) [25], a large-scale, randomized-controlled trial. The previously reported variability in changes in body composition and RMR [26] enabled us to examine the inter-individual variability in the contribution of tissue losses and metabolic adaptations to RMR reduction following weight loss.

## Methods

### Study design

The present investigation is a secondary analysis of data from CALERIE [25], a randomized clinical trial in humans that involved a 25% reduction in energy intake over the course of a 2-year period. CALERIE was chosen because it examined long-term weight loss in a free-living study and the design enabled examination of variability in the causes of RMR reductions secondary to weight loss, as well as changes in body composition and hormonal concentrations. All participants signed an informed consent before study participation. Institutional review boards at Pennington Biomedical Research Center, Washington University Medical Center, and Tufts University oversaw the study and the Duke Clinical Research Institute served as the coordinating center [25]. Despite a common goal of 25% calorie restriction, there was no mandatory diet composition imposed nor specific physical activity required. Dietitians, physicians, and psychologists gave participants individual counseling sessions and an interactive database to support and monitor adherence to calorie restriction prescriptions. Detailed procedures can be found elsewhere [25, 27]. The study was registered at clinicaltrials.gov as NCT00427193.

### Data extraction

Data were obtained via download of the publicly available dataset [28]. Data from baseline, 6 months, and 12 months were chosen for this analysis for several reasons. First, metabolic adaptations are more likely to occur during early weight loss [29, 30]. Second, maximal weight loss in the trial was achieved at month 12, with no significant deviations at later time points [31]. Third, hormonal data, which was needed to confirm the presence of metabolic adaptations, was measured only at baseline and month 12. Finally, later time points at 18 and 24 months had higher attrition.

### Participants

Potential participants were screened for previous eating disorders, significant health problems, recent substantial weight loss and/or participation in CALERIE Phase 1, and use of medication except oral contraceptives. Following initial inclusion, 220 male and female participants 20–50 years of age with a body mass index (BMI) from 22 to 27.9 kg/m^2^ were randomly assigned 2:1 to 25% caloric restriction (*n* = 145) or control group (*n* = 75). For the purpose of our analysis, participants in the non-restricted control groups were excluded as only minimal weight loss was expected. Further, data from participants without complete baseline or 12-month measurements were excluded.

### Assessments

Data used for the present analysis included assessments of body weight, body composition, RMR, and metabolic hormone concentrations. Body weight was assessed every 3 months during a clinical visit using an electric scale (Scale Tronix 5200; Welch Allyn). Body composition was measured using dual-energy x-ray absorptiometry (DXA) via a standardized protocol using a 4500 A, Delphi W, or Discovery A scanner (all Hologic, Waltham, MA) at baseline and months 6 and 12. RMR was measured using indirect calorimetry (Vista-MX metabolic cart; Vacumed, Ventura, CA) at baseline and months 6 and 12. Metabolic hormones, including insulin, leptin, triiodothyronine (T3), and insulin-like growth factor 1 (IGF-1), were assessed from venous blood samples at baseline and month 12.

### Calculations

The extent of changes in RMR attributable to the losses of energy-expending tissues and organs was calculated based on the contribution of the primary organs and tissues contributing to whole-body RMR [16, 32]. Organs and tissues used for this calculation included skeletal muscle, adipose tissue, bone, brain, and inner organs (heart, liver, kidneys). Residual mass was obtained by subtracting each of the organ and tissue masses from total mass. The size of these organs and tissues were determined as previously reported [22, 33]. Skeletal muscle, adipose tissue, bone mass, and brain mass, were assessed from DXA-derived values of lean tissue in the extremities, fat mass, bone mineral content, and skull area, respectively [22, 34]. Internal organs weights were calculated from lean body mass in the trunk [33] (Supplementary Table 1). Metabolic rates for all organ and tissues were calculated by multiplying the size of each organ/tissue with their specific metabolic rate [16]. Predicted RMR was calculated as the sum of the metabolic rates of all eight components. This method has previously been used to quantify adaptive reductions in RMR in various weight loss settings [32, 35] as well as in chronically energy-deficient populations such as anorexia nervosa patients [24] and amenorrheic female athletes [22]. The extent of the metabolic adaptations was subsequently calculated as the difference between changes in measured RMR by indirect calorimetry and changes in predicted RMR [32].

### Statistical analyses

Statistical analyses were performed with R (version 4.0.3, The R Foundation for Statistical Computing). If not labeled otherwise, all data are presented as mean ± standard error of the mean. Changes in outcomes between baseline and months 6 and 12 were assessed using pairwise, paired *T*-tests using the Holm–Bonferroni method.

To determine how reductions in RMR and metabolic adaptations were related to skeletal muscle and adipose tissue losses, linear regression analyses were conducted between outcomes and changes in measured RMR, changes in RMR due to tissue losses, and metabolic adaptations. Associations between metabolic adaptations and measured RMR with changes in adipose tissue and skeletal muscle mass were further assessed using Pearson’s correlation coefficient.

To visualize differences between individuals who lost the most and least of the specific tissues, participants were grouped into quartiles based on their individual changes in skeletal muscle and adipose tissue, respectively, with the lowest (Q1) and highest (Q4) quartiles referring to the participants with the greatest losses and the smallest losses and/or gains in a specific tissue, respectively. Differences in RMR and metabolic adaptations between quartiles were assessed using generalized linear model analyses adjusted for confounders (age, sex, body weight, height, initial BMI, body fat percentage), using Q1 as reference quartiles.

The association between metabolic adaptations and hormonal changes was assessed using Pearson’s correlation coefficient. Statistical significance was considered with a probability of error <5% (*p* < 0.05).

## Results

The present analysis included 77 women and 32 men (*n* = 109) with complete data at baseline and 12 months (Table 1).

**Table 1:** Baseline characteristics of the 109 participants included in the analysis.

	All participants (*n* = 109)	Female participants (*n* = 77)	Male participants (*n* = 32)
Age (yr)	37.8 ± 7.4	36.4 ± 7.3	41.0 ± 6.6
Body Weight (kg)	71 ± 8.5	67.5 ± 6.2	79.6 ± 7.0
Height (cm)	168.2 ± 7.8	165.3 ± 6.7	175.2 ± 5.4
Body Mass Index (kg/m^2^)	25.0 ± 1.7	24.7 ± 1.6	25.9 ± 1.5
Body Fat (%)	33.0 ± 5.9	35.9 ± 4.2	26.0 ± 2.7

**Fig. 1:**
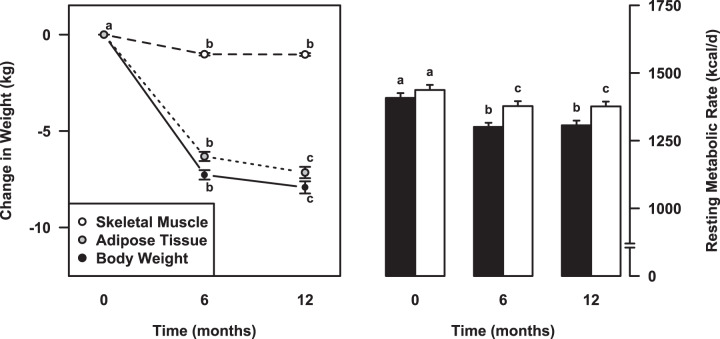
Changes in body weight, skeletal muscle, adipose tissue, and resting metabolic rate over the course of 12 months of calorie-restricted weight loss. Left: Changes in body weight (closed symbols), skeletal muscle (open symbols), and adipose tissue (gray symbols) over the course of the first 12 months of calorie-restricted weight loss. Right: Changes in measured (black bars) and predicted (white bars) resting metabolic rate over the course of the first 12 months of calorie-restricted weight loss.) Data points with different letters are significantly different from one another (*p* < 0.001).

Body weight declined by 7.3 ± 0.2 kg (*p* < 0.001) from baseline to 6 months and declined by an additional 0.7 ± 0.2 kg (*p* < 0.001) from 6 to 12 months, resulting in a total weight loss of 8.0 ± 0.3 kg (*p* < 0.001) (Fig. 1). Among the RMR-contributing tissues and organs, meaningful changes occurred in skeletal muscle from baseline to 6 months (−1.0 ± 0.7 kg, *p* < 0.001) with no further losses from 6 to 12 months (*p* = 0.83; Table 2). Reductions in adipose tissue occurred from baseline to 6 months (−6.3 ± 0.2 kg; *p* < 0.001) and further from 6 to 12 months (*p* < 0.001) for a total loss of 7.2 ± 3.0 kg. No or only minimal changes were observed for brain, inner organ, bone and residual mass (Table 2).

**Table 2:** Changes in the size of tissues and organs contributing to resting metabolic rate.

	Baseline	Month 6		Month 12	
	Mass (kg)	Mass (kg)	Change	Mass (kg)	Change
Skeletal muscle	23.2 ± 5.2	22.2 ± 4.9	−1.0 ± 0.7^***^	22.2 ± 5.0	−1.0 ± 0.8^***^
Adipose tissue	27.5 ± 4.9	21.1 ± 4.8	−6.3 ± 2.5^***^	20.3 ± 4.6	−7.2 ± 3.1^***,†††^
Brain	1.5 ± 0.2	1.5 ± 0.2	0.0 ± 0.0	1.5 ± 0.2	0.0 ± 0.0
Inner organs	2.0 ± 0.3	1.9 ± 0.3	−0.1 ± 0.1^***^	1.9 ± 0.3	−0.1 ± 0.1^***^
Bone	4.4 ± 0.7	4.5 ± 0.7	0.0 ± 0.1^**^	4.5 ± 0.7	0.0 ± 0.1
Residual mass	13.0 ± 2.9	13.1 ± 2.7	0.1 ± 0.7	13.3 ± 2.6	0.3 ± 0.9^***,†††^

**, ***p < 0.01, 0.001 vs. month 0, ^†††^*p* < 0.001 vs. month 6.

At baseline, there was no difference between measured and predicted RMR (1408 ± 18 kcal/d vs. 1437 ± 19 kcal/d). Measured RMR decreased by 101 ± 12 kcal/d (−7.6%, *p* < 0.001) from baseline to 6 months, and there was no further decline from 6 to 12 months (*p* = 0.59; Fig. 1). Predicted RMR was reduced by 60 ± 3 kcal/d (−4.2%, *p* < 0.001) from baseline to 6 months, with no further decline from 6 to 12 months (*p* = 0.58). Per our operational definition, the additional 40 ± 11 kcal/d reduction in measured RMR were attributed to metabolic adaptations.

**Fig. 2:**
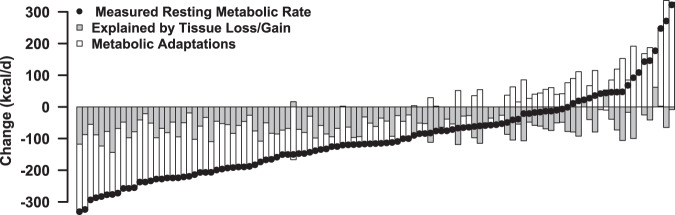
Individual changes in measured resting metabolic rate, resting metabolic rate predicted from changes in organ and tissues, and metabolic adpatations. Contribution of tissue losses and gains (gray bars) and metabolic adaptations (white bars) to the individual changes in resting metabolic rate measured by indirect calorimetry (black diamond) in response to caloric restriction.

Individual changes in measured RMR, RMR predicted from changes in organ and tissues, and metabolic adaptations are shown in Fig. 2. Following the intervention, 83% of participants experienced a reduction in RMR. Of the participants who experienced a reduction in RMR, the reduction in RMR was between 0 and 100 kcal/d in 27%, between 100 and 200 kcal/d in 33%, between 200 and 300 kcal/d in 21%, and >300 kcal/d in 2%. In 61%, metabolic adaptations contributed more substantially to the change in RMR than tissue and organ changes, and 33% of participants experienced positive metabolic adaptations, i.e., an increase in RMR despite tissue losses.

**Fig. 3:**
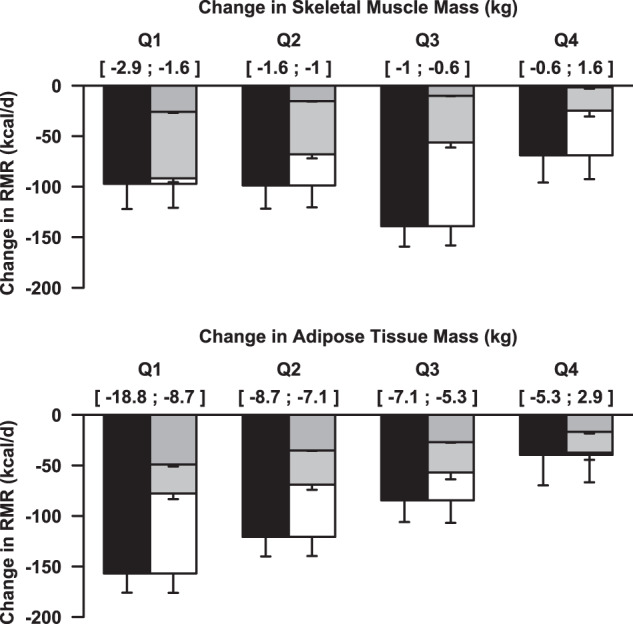
Changes in Resting Metabolic Rate (RMR) based on the amount of skeletal muscle and adipose tissue loss from baseline to month 12. Black bars depict changes in RMR measured by indirect calorimetry, dark gray bars depict changes in RMR predicted from skeletal muscle mass (top) or adipose tissue (bottom), light gray bars depict changes in RMR predicted from the other organ and tissues, and white bars depict metabolic adaptations. All comparisons are adjusted for age, sex, weight, height, initial body mass index (kg/m^2^), and body fat percentage.

Upon examination of the relationship between tissue losses and the reductions in RMR and metabolic adaptations, there were no discernible associations between changes in skeletal muscle mass and measured RMR (*p* = 0.16) nor between changes in skeletal muscle mass and metabolic adaptations (*p* = 0.36) (Supplementary Fig. 1). A significant linear relationship was observed between changes in adipose tissue mass and measured RMR (17 kcal/kg; *p* < 0.001) and between changes in adipose tissue mass and metabolic adaptations (12 kcal/kg; *p* < 0.001) (Supplementary Fig. 2). After stratifying participants into quartiles based on losses in skeletal muscle and adipose tissue mass, there was no discernible difference between changes in measured RMR and the amount of skeletal muscle lost (Fig. 3). In Q1, where individuals experienced the greatest loss of skeletal muscle mass (1.6–2.9 kg), skeletal muscle loss only accounted for 26.7% of the RMR reduction. Reductions in other organs and tissues accounted for another 67.7%, resulting in a reduction in RMR that was almost completely (94.4%) accounted for by all tissue losses. As less skeletal muscle was lost, the proportion of RMR reduction accounted for by total tissue losses declined (*p* < 0.001). As a result, the proportion of metabolic adaptations accounting for the overall reduction in RMR increased steadily from Q1 (5.6%) to Q4 of skeletal muscle (63.9%), although this increase failed to achieve statistical significance (*p* = 0.11).

When dividing participants into quartiles based on adipose tissue losses, the reduction in measured RMR declined as less adipose tissue was lost, as did the reduction in predicted RMR from tissue losses (Fig. 3). In individuals with the greatest loss of adipose tissue (Q1: 8.7–18.8 kg), adipose tissue loss accounted for 31.3% of RMR reduction and the loss of other tissues accounted for another 18.3%, resulting in total tissue losses accounting for 49.6% of the reduction in RMR. The proportion of RMR reduction explained by all tissue and organ losses increased proportionally from Q2 (57.2%) to Q4 (94.5%). Consequently, metabolic adaptations, which decreased in their contribution to RMR reduction from Q1 (50.4%) to Q4 (5.5%), were positively associated with adipose tissue losses (*p* < 0.001, *b* = 11.9 kcal/kg adipose tissue).

**Fig. 4:**
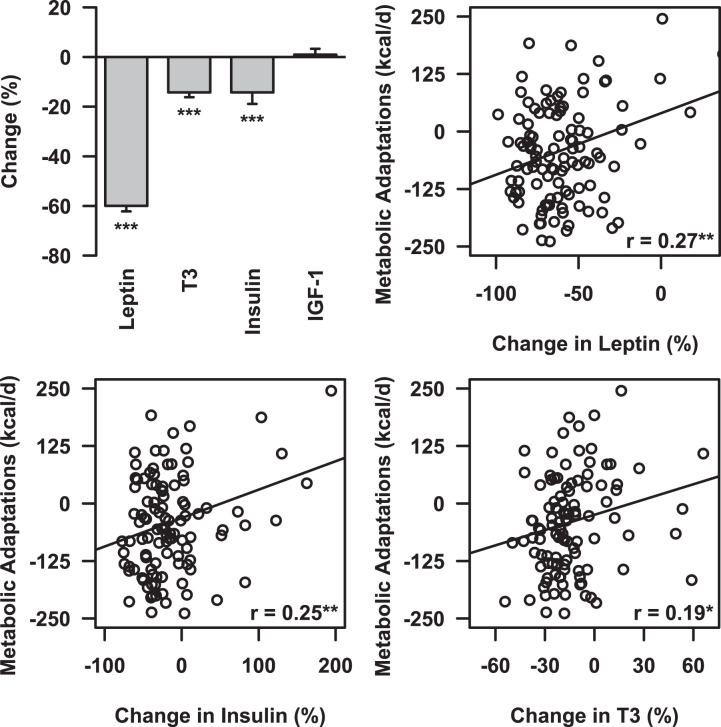
Reductions in metabolic hormones over the course of 12 months of calorie-restricted weight loss. Reductions in leptin, trioiodothyronine (T3), insulin, and insulin-like growth factor-1 (IGF-1) (top left) and correlations between leptin (top right), insulin (bottom left), and T3 (bottom right) with metabolic adaptations. ^*^p < 0.05; ^**^p < 0.01.

There were significant reductions in metabolic hormones after 12 months, with the exception of IGF-1 (Fig. 4). The largest decline occurred in leptin, which decreased by 59.9 ± 2.2% (*p* < 0.001). T3 (−14.3 ± 2.0%) and insulin (−14.3 ± 4.6%) were also significantly reduced (both *p* < 0.001). Reductions in leptin (*r* = 0.27, *p* < 0.01), T3 (*r* = 0.19, *p* < 0.05), and insulin (*r* = 0.25, *p* < 0.01) were all significantly correlated with metabolic adaptations (Fig. 4).

## Discussion

The present retrospective analysis of data from a large, well-controlled randomized trial confirms that RMR is reduced by a magnitude of ~100 kcal (~7%) in response to weight loss of ~11% achieved through caloric restriction in healthy normal weight and overweight individuals. On average, only 60% of the total reduction in RMR were explained by losses of energy-expending tissues, while the remaining 40% of RMR reduction can be attributed to metabolic adaptations. However, there was substantial variability between participants in RMR changes as well as in the contributions of tissue losses and metabolic adaptations to RMR changes.

The observed RMR reduction by 7% is similar to previous, albeit shorter and more aggressive weight loss studies, such as in individuals who lost 9.6 kg (~10% of initial body weight) and exhibited a 9% reduction in RMR [36] after 8 weeks on a very-low-energy diet (500 kcal/day). Similarly, a 10% reduction in RMR was reported in participants who lost 8.9 kg (~9% of initial body weight) after consuming 550–660 kcal/day for 4 weeks [37].

Upon analysis of tissue-specific weight loss in CALERIE, we observed that the primary components lost during the intervention were skeletal muscle and adipose tissue, while the remaining organs and tissues were largely preserved. The selective loss of these two components aligns with a previous examination of the specific composition of FFM loss during weight loss, which observed no disproportionate loss of high-metabolically active organs when compared to skeletal muscle [17]. While bone mineral density can be reduced following weight loss, bone mass was not lowered in the present study, which could be attributed to the slow rate of weight loss when compared to more restrictive weight loss reporting bone loss and the provision of calcium supplementation [38]. Consequently, we stratified participants into quartiles based on skeletal muscle losses, rather than examining it along with the other components of FFM (e.g., bone, brain, inner organs) that were largely preserved. We observed that individuals who lost the greatest amount of skeletal muscle exhibited a reduction in RMR that was only ~25% explained by skeletal muscle itself, but almost entirely explained by the reduction of all energy-expending tissues (95%). As the other components of FFM were relatively preserved, the other 65% of the RMR reduction explained by tissues is attributable to the large losses in adipose tissue. The contribution of all tissue losses to the reduction observed in RMR became less prominent in Q2–Q4 of skeletal muscle loss, increasing the contribution of metabolic adaptations to the reduction in RMR across Q2–Q4 despite improved skeletal muscle preservation.

Our results differ from other studies reporting that reductions in RMR were almost entirely explained by reductions in FFM, such as the 6% reduction in RMR following 3 weeks of a low-energy diet and 1.9 kg reduction in FFM [39] or the even more substantial FFM losses (−3.4 kg) occurring after 10-16 weeks of caloric restriction [14], which resulted in an unaltered RMR when expressed relative to FFM. However, it is important to note that these studies looked at overall FFM, rather than the specific component that is primarily lost (i.e., skeletal muscle) and its associated expenditure. Aside from bone, skeletal muscle expends substantially less energy (~13 kcal/kg/d) than the other organs that make up FFM (~200–450 kcal/kg/d). Because our approach examined the loss of each tissue and organ, we were able to calculate the direct energy footprint associated with the loss of each tissue. When taking its lower tissue-specific energy expenditure relative to the other higher expenditure components of FFM into account, it is therefore not surprising that skeletal muscle losses did not explain all RMR reductions.

While tissue changes explained slightly more than half of the RMR reduction following weight loss (60%), Fig. 2 suggests the high inter-individual variation in RMR occurred as a result of metabolic adaptations. RMR reductions secondary to metabolic adaptations have been frequently observed after weight loss [19, 29, 30, 40, 41], and are understood as a sign of the suppression of non-vital processes to decrease energy expenditure, which ultimately attenuates weight loss [42, 43]. The extent of metabolic adaptations in the present study was quantified at ~40 kcal/d following 12 months of a 25% caloric deficit. Using the same method, Müller et al. quantified metabolic adaptations of ~70 kcal/d in a shorter and more restrictive setting of a 50% energy deficit over only 7 days [19].

Our analysis further indicated that the extent of metabolic adaptations in the present study was strongly related to the amount of adipose tissue lost. This positive association between adipose loss and metabolic adaptations is in agreement with previous studies in individuals with obesity undergoing gastric bypass surgery or an intensive weight loss program [21]. Both sets of participants lost ~40–50 kg weight, but adipose tissue losses differed. Yet the extent of metabolic adaptations appeared to be commensurate to adipose tissue losses. When expressing metabolic adaptations relative to adipose tissue losses, both groups experienced metabolic adaptations in the same range (201 kcal/26.5 kg = 7.6 kcal/kg; 419 kcal/47.9 kg = 8.7 kcal/kg) [21] as what we observed in Q1 of adipose tissue losses (79 kcal/10.9 kg = 7.2 kcal/kg tissue). To further corroborate the presence of metabolic adaptations, the extent of metabolic adaptations in our sample was strongly correlated to changes in circulating concentrations of the key energy-sensing hormones leptin and T3. While confirming the associative nature of reductions in metabolic hormones and metabolic adaptations, our data are strengthened by findings that exogenous administration of leptin and T3 at least partially reverse reductions in energy expenditure following weight loss [44]. However, it remains to be tested whether metabolic hormone replacement attenuates adaptive reductions in the metabolic activity of the remaining tissues and organs, which could make it an interesting strategy to combat the metabolic adaptations leading to RMR reduction.

Despite multiple literature reports of metabolic adaptations following weight loss, it is important to note that there is no gold standard method for its direct measurement. Metabolic adaptations represent the difference between measured and predicted RMR. To optimize its quantification, we utilized DXA data, which enabled more specific quantification of energy-expending tissues and organs to improve the prediction of RMR [34]. The equations and coefficients used in the present study were previously established and validated in examinations of underweight, normal weight, and individuals with obesity [33] across adulthood [45], in several weight-loss settings [32, 35], and for the quantification of metabolic adaptations in non-obese men [19]. While some of these studies estimated inner organ masses using magnetic resonance imaging, we remain confident that this present method of calculating metabolic adaptations was able to effectively compare the extent of metabolic adaptations across the intervention. To test the predictability of our model, we compared measured and predicted RMR at baseline in a presumed state of energy balance and found no systematic difference between measured and predicted RMR (*p* = 0.27).

While the present analysis describes the contribution of changes in energy-expending tissues and organs and metabolic adaptations to the reduction in RMR in a large caloric restriction trial, it was conducted in non-obese individuals, whose weight loss requirements are not the same as individuals with obesity. However, given that changes in non-adipose tissues tend to be greater in leaner individuals [46], the non-obese study population allowed us to examine a wider spectrum of body composition changes and ascertain how their contribution to RMR reductions during weight loss varies depending on whether they are lost or preserved. Further, the way in which caloric restriction was attained was not tightly controlled. However, our analysis focused on the two additive components of RMR reduction occurring secondary to weight loss, irrespective of how weight loss was achieved.

## Conclusions

Our analysis demonstrates that RMR is inevitably reduced after weight loss in healthy normal weight and overweight individuals and that this reduction occurs through a combination of the loss of energy-expending tissues and metabolic adaptations. As hypothesized, the loss of energy-expending tissues—predominantly skeletal muscle and adipose tissue—contributed to the reduction in RMR, although on average for only ~60% (60 ± 3 kcal/d), leaving the remaining 40% of the RMR reduction (40 ± 11 kcal/d) attributable to metabolic adaptations. More importantly, the contribution of tissue losses and metabolic adaptations to overall RMR reduction was highly variable between individuals. Contrary to common belief, there was no discernible relationship between the loss of skeletal muscle, the primary lean tissue component that is lost during weight loss, and reductions in RMR. Conversely, the loss of adipose tissue was related to reductions in RMR and metabolic adaptations, whereby metabolic adaptations were greatest in individuals who lost the most adipose tissue. Given the differential impact of these components to RMR reduction following weight loss, future research should examine whether the preservation of the tissues or their metabolic activity yields differential results toward RMR reductions and weight maintenance and whether more personalized strategies addressing the specific cause of the RMR reduction may help maximize weight loss and prevent weight regain.

## Supplementary information

Supplementary Figure and Table Legends

Supplementary Table 1

Supplementary Figure 1

Supplementary Figure 2
