# Supplementary material for: Tissue losses and metabolic adaptations both contribute to the reduction in resting metabolic rate following weight loss
Source: Int J Obes (Lond). 2022 Feb 18;46(6):1168–75. doi: 10.1038/s41366-022-01090-7 (PMC9151388; doi:10.1038/s41366-022-01090-7)
Supplement: Supplementary file 1 — Supplementary Figure and Table Legends [file 41366_2022_1090_MOESM1_ESM.pdf]

Supplementary Figure and Table Legends

Supplementary Figure 1. Correlation between the change in skeletal muscle mass (kg) with changes in measured resting metabolic rate, changes in resting metabolic rate predicted from changes in skeletal muscle mass, changes in resting metabolic rate predicted from changes in other organs and tissues, and the extent of metabolic adaptations.

Supplementary Figure 2. Correlation between the change in adipose tissue mass (kg) with changes in measured resting metabolic rate, changes in resting metabolic rate predicted from changes in adipose tissue mass, changes in resting metabolic rate predicted from changes in other organs and tissues, and the extent of metabolic adaptations.

Supplementary Table 1. Calculations to Predict Resting Metabolic Rate from Dual X-Ray Absorptiometry Scans
