# Supplementary material for: Tissue losses and metabolic adaptations both contribute to the reduction in resting metabolic rate following weight loss
Source: Int J Obes (Lond). 2022 Feb 18;46(6):1168–75. doi: 10.1038/s41366-022-01090-7 (PMC9151388; doi:10.1038/s41366-022-01090-7)
Supplement: Supplementary file 2 — Supplementary Table 1 [file 41366_2022_1090_MOESM2_ESM.pdf]

Supplementary Table 1. Calculations to Predict Resting Metabolic Rate from Dual X-Ray Absorptiometry Scans

Organ/Tissue	Mass (kg) [a-c]	Expenditure (kcal/kg/d) [d]
Skeletal Muscle	Men: (1.13 * Lean $Tissue_{ARMS+LEGS}$ (kg)) – (0.02 * age(yr))+1.58	13
	Women: (1.13 * Lean $Tissue_{ARMS+LEGS}$ (kg)) – (0.02 * age(yr))+.97	
Adipose Tissue	1.18 * Fat Mass (kg)	4.5
Bone	1.85 * Bone Mineral Content (kg)	2.3
Brain	Men: 0.005 * Head Area (cm^2) + 0.44	240
	Women: .005 * Head Area (cm^2) + 0.24	
Heart	0.012 * Lean Mass Trunk $(kg)^{1.0499}$	441
Liver	0.0778 * Lean Mass Trunk $(kg)^{0.9277}$	201
Kidneys	0.0165 * Lean Mass Trunk $(kg)^{0.9306}$	441
Residual Mass	Total Mass (kg) - ∑ Mass (Skeletal Muscle + Adipose Tissue + Bone + Brain + Heart + Liver + Kidneys)	6.9

[a] Bosy-Westphal A, Reinecke U, Schlorke T, Illner K, Kutzner D, Heller M, et al. Effect of organ and tissue masses on resting energy expenditure in underweight, normal weight and obese adults. Int J Obes Relat Metab Disord. 2004;28(1):72-9.
[b] Koehler K, Williams NI, Mallinson RJ, Southmayd EA, Allaway HC, De Souza MJ. Low resting metabolic rate in exercise-associated amenorrhea is not due to a reduced proportion of highly active metabolic tissue compartments. American journal of physiology Endocrinology and metabolism. 2016;311(2):E480-7.
[c] Hayes M, Chustek M, Wang Z, Gallagher D, Heshka S, Spungen A, et al. DXA: potential for creating a metabolic map of organ-tissue resting energy expenditure components. Obes Res. 2002;10(10):969-77.
[d] Elia M. Organ and tissue contribution to metabolic rate IN Energy metabolism: Tissue Determinant and Cellular Corrolaries. New York, NY: Raven Press; 1992.
