# Supplementary figures and images for: Tissue losses and metabolic adaptations both contribute to the reduction in resting metabolic rate following weight loss
Source: Int J Obes (Lond). 2022 Feb 18;46(6):1168–75. doi: 10.1038/s41366-022-01090-7 (PMC9151388; doi:10.1038/s41366-022-01090-7)

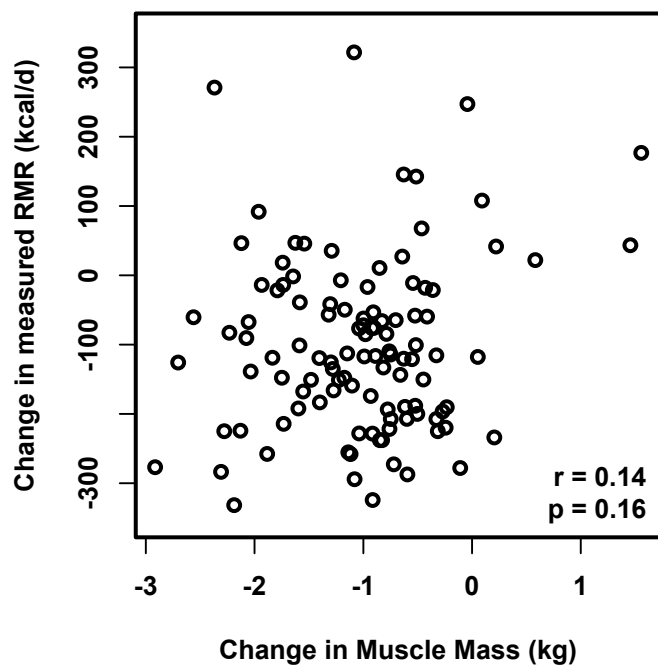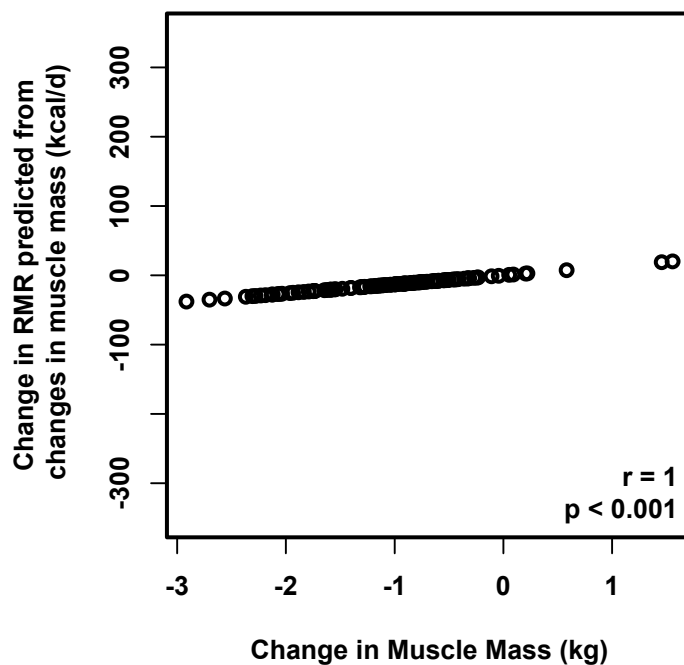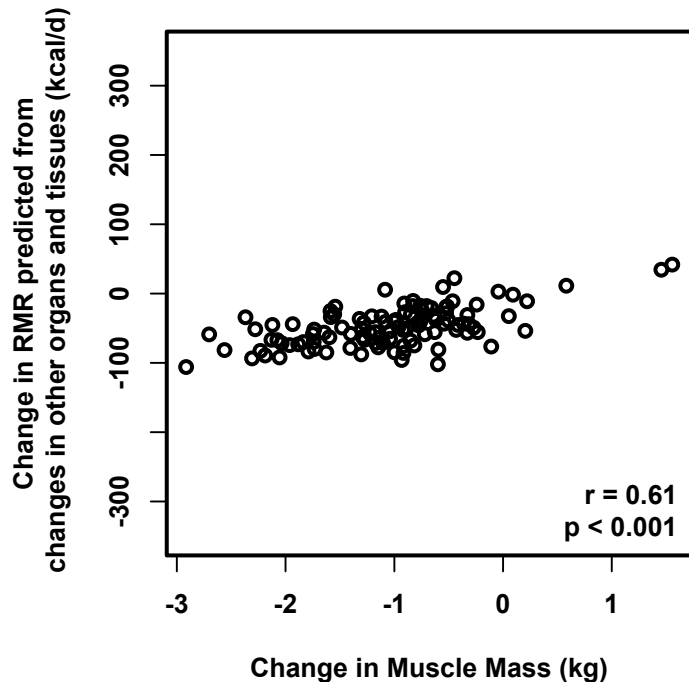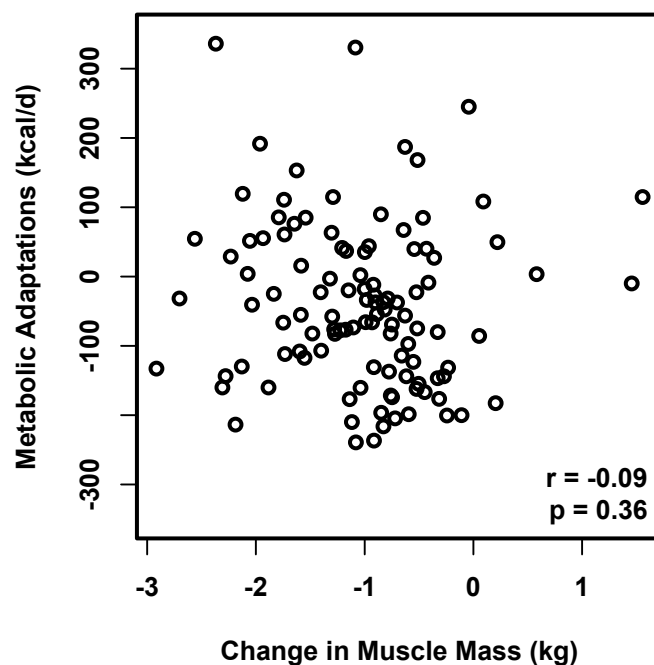

Supplement: Supplementary file 3 — Supplementary Figure 1 [file 41366_2022_1090_MOESM3_ESM.pdf]

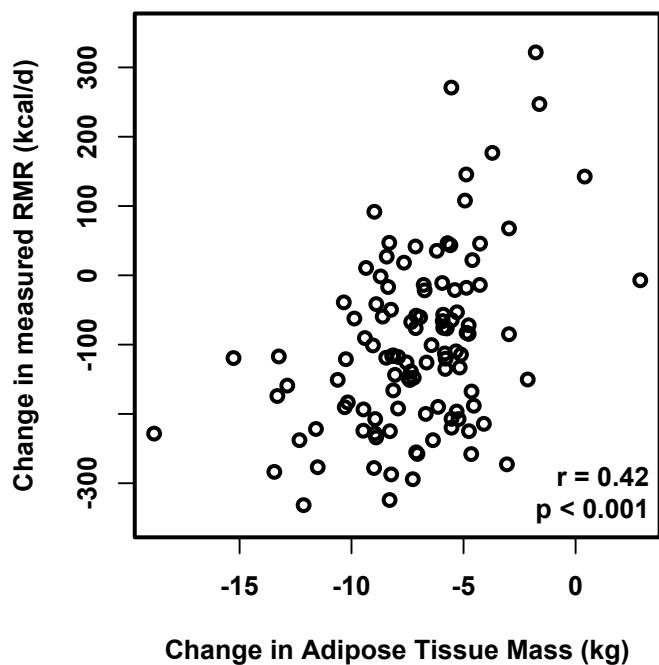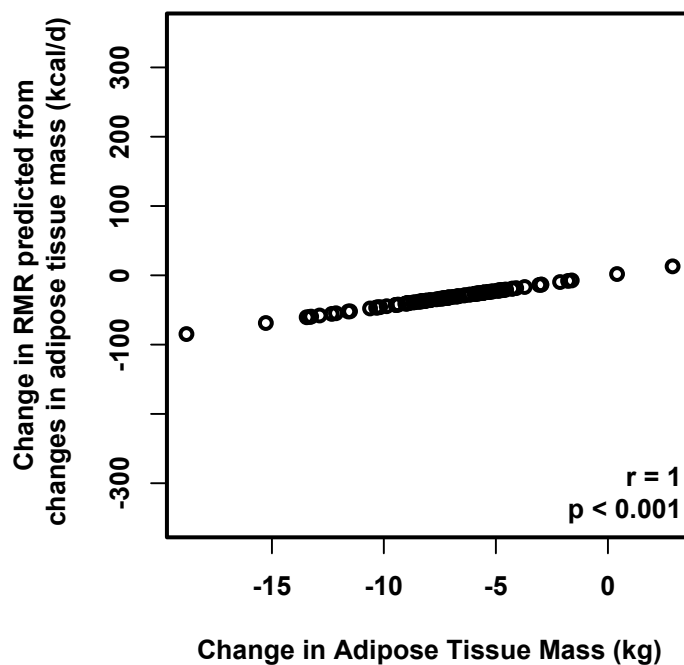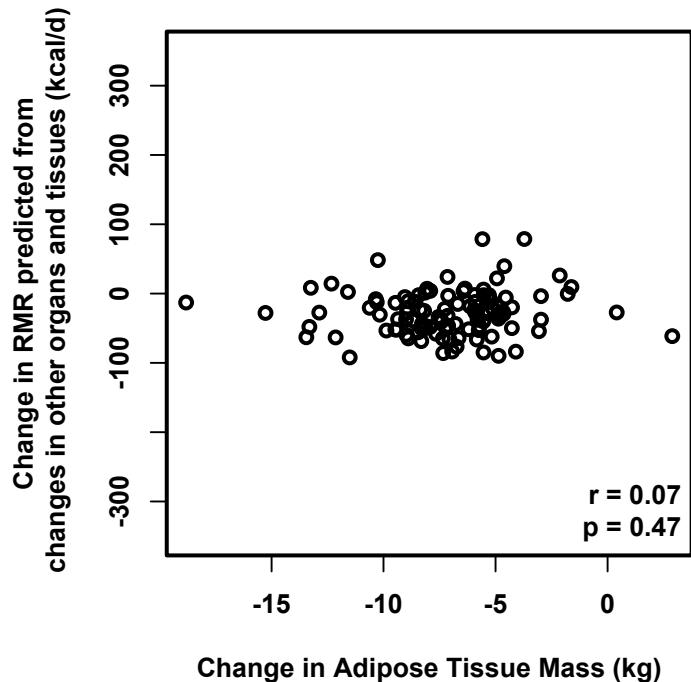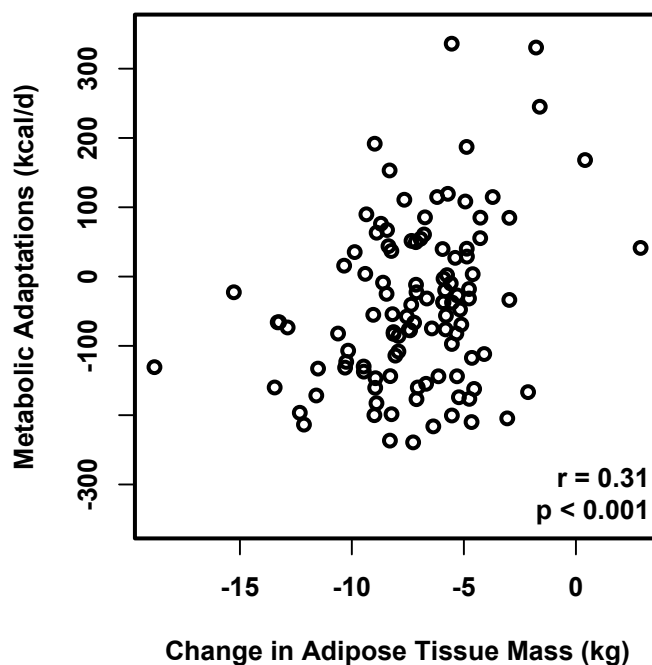

Supplement: Supplementary file 4 — Supplementary Figure 2 [file 41366_2022_1090_MOESM4_ESM.pdf]
